# Predictors of job satisfaction and intention to stay in the job among health-care providers in Uganda and Zambia

**DOI:** 10.1093/intqhc/mzab128

**Published:** 2021-09-09

**Authors:** min kyung kim, catherine arsenault, Lynn m Atuyambe, Margaret e Kruk

**Affiliations:** Department of Global Health and Population, Harvard T.H. Chan School of Public Health, 655 Hungtington Ave, Boston, MA 02115, USA; Department of Global Health and Population, Harvard T.H. Chan School of Public Health, 655 Hungtington Ave, Boston, MA 02115, USA; Department of Community Health and Behavioral Sciences, Makerere University School of Public Health, New Mulago Hill road, Mulago, Kampala, Uganda; Department of Global Health and Population, Harvard T.H. Chan School of Public Health, 655 Hungtington Ave, Boston, MA 02115, USA

**Keywords:** job satisfaction, intention to stay, health-care provider, Uganda, Zambia

## Abstract

**Background:**

A shortage of competent health-care providers is a major contributor to poor quality health care in sub-Saharan Africa. To increase the retention of skilled health-care providers, we need to understand which factors make them feel satisfied with their work and want to stay in their job. This study investigates the relative contribution of provider, facility and contextual factors to job satisfaction and intention to stay on the job among health-care providers who performed obstetric care in Uganda and Zambia.

**Methods:**

This study was a secondary analysis of data from a maternal and newborn health program implementation evaluation in Uganda and Zambia. Using a Likert scale, providers rated their job satisfaction and intention to stay in their job. Predictors included gender, cadre, satisfaction with various facility resources and country. We used the Shapley and Owen decomposition of *R*^2^ method to estimate the variance explained by individual factors and groups of factors, adjusting for covariates at the facility and provider levels.

**Results:**

Of the 1134 providers included in the study, 68.3% were female, 32.4% were nurses and 77.1% worked in the public sector. Slightly more than half (52.3%) of providers were strongly satisfied with their job and 42.8% strongly agreed that they would continue to work at their facility for some time. A group of variables related to facility management explained most of the variance in both job satisfaction (37.6%) and intention to stay (43.1%). Among these, the most important individual variables were satisfaction with pay (20.57%) for job satisfaction and opinions being respected in the workplace (17.52%) for intention to stay. Doctors reported lower intention to stay than nurses. Provider demographics and facility level and ownership (public/private) were not associated with either outcome. There were also differences in job satisfaction and intention to stay between Ugandan and Zambian health-care providers.

**Conclusion:**

Our study suggests that managers play a crucial role in retaining a sufficient number of satisfied health-care providers providing obstetric care in two sub-Saharan African countries, Uganda and Zambia. Prioritizing and investing in health management systems and health managers are essential foundations for high-quality health systems.

## Introduction

The health-care provider shortage is a major impediment to providing high-quality health care in sub-Saharan Africa [1, 2]. The World Health Organization estimates a shortage of 12.9 million skilled health professionals by 2035, with the greatest shortfall in Africa and Southeast Asia [3]. This shortage has been exacerbated by brain drain in which providers from low-income countries emigrate to higher-income countries for better paid and more advanced professional opportunities. For example, it is estimated that around 7000 African nurses have registered to work in the United Kingdom since 2001 [1].

Job satisfaction, ‘a gratifying emotional state from the appraisal of one’s job’ [4], in addition to being an intrinsically valuable objective of any job, is instrumental in preventing health-care providers from leaving their current workplace. Several studies have shown that more satisfied health-care providers are more likely to remain in their current job [5–7]. Thus, job satisfaction has received a great deal of attention in research because of its potential effect on retaining health-care providers.

Factors related to the provider, the facility or the context can affect job satisfaction. For example, the length and type of training providers experience is linked to levels of job satisfaction among health-care providers in low-income countries [8, 9]. In addition, facility and contextual factors such as availability of functional equipment and job prospects in rural and urban areas have been identified as important contributors in keeping providers feeling satisfied in several sub-Saharan African countries [10–13]. Meanwhile, staff shortages, difficulty in accessing transport for work and inadequate support from supervisors contributed to job dissatisfaction among health-care workers in studies in Kenya and Ghana [9, 11].

However, little is known about differences in job satisfaction and intention to stay across provider cadres. Previous studies have been mostly focused on midwives [7, 14] and nurses [5, 6]. Understanding how these outcomes vary across provider cadres is needed to obtain a more comprehensive picture of the drivers of job satisfaction and intention to stay.

The purpose of this study is to investigate the relative contribution of provider, facility and contextual factors on job satisfaction and intention to stay in their job among doctors, nurses and other clinicians who performed obstetric care in Uganda and Zambia. Since 2012, total fertility rates have slightly decreased in Uganda (from 6.2 in 2011 to 5.4 in 2016) and Zambia (from 6.2 in 2007 to 4.7 in 2018) [15, 16]. The proportion of women giving birth in health facilities have increased considerably in Uganda (from 57% in 2011 to 73% in 2016) and Zambia (from 52% in 2007 to 84% in 2018) [15, 16]. Thus, our results can provide useful insights to designing policies to improve health-care provider satisfaction and retain health-care professionals in sub-Saharan Africa.

## Methods

### Study setting and sample

This study uses data from the evaluation of the Saving Mothers and Giving Life (SMGL) intervention [17]. The SMGL intervention was implemented in Uganda and Zambia from January to June 2012. This intervention was implemented in four districts—Kabarole, Kamwenge, Kibaale and Kyenjojo in Uganda and Mansa, Lundazi, Nyimba and Kalomo in Zambia. These districts were primarily rural, with a largely agricultural workforce. The districts were selected based on a high maternal mortality ratio, a low facility delivery rate and a high health-care provider shortage. The purpose of the intervention was to improve the quality of care during labor and delivery. The main program activities included are as follows: (i) providing new equipment and supplies, (ii) mentoring providers with experienced clinicians, (iii) upgrading the maternity wards and (iv) building new operating theaters to perform cesarean sections in Uganda. The core inputs and activities of the SMGL have been published elsewhere [18].

The design of the evaluation study was a quasi-random post-test-only comparison group; details of the evaluation have been previously published [19]. The data for the evaluation were collected from May 2013 to July 2013. The evaluators selected two comparison districts per country—Masinid and Kiryandongo in Uganda and Kapiri Mposhi and Kabwe in Zambia—based on their similarity with the intervention districts in terms of geography, health system infrastructure, health system utilization, and morbidity and mortality. Health facilities were included based on their delivery volume, urban/rural location, and whether they offer comprehensive emergency obstetric and newborn care.

### Instruments

During the SMGL evaluation, three questionnaires were administered to maternal health-care workers: an obstetric knowledge test, a confidence questionnaire (gauging their reported confidence in performing 26 common obstetric tasks) and a job satisfaction questionnaire. The three instruments were pilot tested in non-study districts among providers in Uganda and Zambia prior to the start of data collection and revised accordingly. All clinicians available in the facility on the days of the study were invited to complete the survey. Eligible health-care providers included all clinicians (i.e. doctors, nurses, midwives, nurse assistants and clinical officers) who provided obstetric care. To maximize the response rate, interviewers returned to the facility on different days to accommodate health-care providers’ schedules.

The job satisfaction questionnaire measured the workers’ satisfaction with various aspects of their work, including the providers’ opinions related to their work environment (such as availability of functional equipment and adequate clinical supervision), as well as overall satisfaction about their current work and their intention to stay in their current health facility for some time (**Supplementary Appendix A**). The survey was administered in person in English. SurveyCTO software was used to enter the data on Galaxy Nexus t computers. Information on providers’ demographics and characteristics of facilities were also collected. Results from the two other instruments—knowledge test and confidence assessment—have been published elsewhere [20].

### Measures

#### Outcomes

We selected two provider outcomes: job satisfaction and intention to stay. Job satisfaction was assessed using the question: ‘In general, I am satisfied with this job.’ Intention to stay in the job was assessed using the question: ‘If it were up to me, I would continue to work for this hospital/clinic for quite some time.’ Both questions were measured on a 4-point Likert scale—strongly disagree, somewhat disagree, somewhat agree and strongly agree. We treated them as continuous variables for our analysis ranging from 1 for strongly disagree to 4 for strongly agree.

#### Covariates

We included six categories of covariates at the provider and facility levels known to influence provider intention to stay and job satisfaction: (i) demographics, (ii) cadre, (iii) facility characteristics, (iv) perceptions on the work environment related to inputs, (v) perceptions on the work environment related to management and (vi) context of the facility.

Demographics included age, gender and on-site training. Age was treated as a continuous variable. To account for non-linearity, a quadratic term for age was also included. Amount of training received in the past year was measured as the total number of days during which providers reported receiving on-site trainings.

Cadre included seven types of providers based on the length of training: (i) nurse assistant, (ii) enrolled nurse, (iii) enrolled midwife, (iv) registered nurse, (v) registered midwife, (vi) clinical officer and (vii) general doctor, doctor specialist and medical licentiate. Nurse assistants are trained for about 6 months and exist only in Uganda. Enrolled nurses and enrolled midwives are trained for 2–3 years. Enrolled nurses are similar to licensed practical nurses in the United States. Registered nurses and registered midwives are trained for 3–4.5 years. Clinical officers receive 3 years of training. Doctors are typically trained for 5–7 years. Medical licentiates are clinical officers who received additional training so that they can perform several tasks that a doctor would typically perform [21]. Given the similar years of training, medical licentiates were included in the same group as doctors.

Facility characteristics included facility type and public vs. private ownership. Facility type was based on availability of services and included basic emergency obstetric and neonatal care (BEmONC) facilities and comprehensive obstetric and neonatal care (CEmONC) facilities. BEmONC facilities perform seven basic functions: (i) administration of parenteral antibiotics, (ii) administration of uterotonic drugs for active management of the third stage of labor and prevention of postpartum hemorrhage, (iii) use of parenteral anticonvulsants for the management of preeclampsia/eclampsia, (iv) manual removal of placenta, (v) removal of retained products, (vi) assistance of vaginal delivery and (vii) basic neonatal resuscitation [22]. CEmONC facilities must perform the seven basic BEmONC functions in addition to cesarean sections and blood transfusion [22]. Private ownership included both for-profit and not-for-profit facilities.

Two categories of covariates related to the work environment—inputs and management—were included. The input category grouped variables related to infrastructure, staffing and pay and included three variables measuring providers’ opinion on the functioning equipment and infrastructure to perform their duties, the level of staffing and whether they were satisfied with their current pay compared to similar jobs in other organizations. The management category included four variables measuring provider’s opinion on clinical supervision, whether their workload was manageable, whether their facilities provided adequate in-service (continuing) education to improve their clinical skills and whether they felt like their opinions were respected at work. Questions related to inputs and management of work environment were measured in a 4-point Likert scale—strongly disagree, somewhat disagree, somewhat agree and strongly agree. Providers were assigned 0 point for strongly disagree and somewhat disagree and 1 point for strongly agree and somewhat agree.

Context covariates included urban/rural location, intervention/control districts and country (Uganda or Zambia).

#### Statistical analysis

In order to estimate the proportion of the variance in provider satisfaction and intention to stay explained by each covariate and group of covariates, we used the Shapley and Owen decomposition of *R*^2^ method using the *rego* command in Stata. This method decomposes the *R*^2^ (the ratio of the explained sum of squares to the total sum of squares) of an ordinary least square model indicated by Shapley and Owen values [23]. These values are equivalent to partial *R*^2^ and allow us to assess the explanatory power of individual regressors or groups of regressors in addition to the full model *R*^2^. The unit of analysis was the provider, and the standard errors were adjusted for clustering at the facility level. We also looked at unadjusted correlations between the two outcome variables and the work environment covariates using Pearson’s correlation coefficient. All analyses were conducted in November 2019 using Stata SE version 16.0 (StataCorp, College Station, TX).

#### Ethics

Since this study was a secondary analysis of de-identified data, it was deemed to be non-human subjects research under the Harvard T.H. Chan School of Public Health Institutional Review Board (IRB) policy. The original study was approved by IRB at Columbia University in the United States, Makerere University School of Public Health and the National Council for Science Technology in Uganda, and ERES (Excellence in Research Ethics and Science) Converge Research Ethics Committee and Ministry of Health in Zambia [19]. Consent was obtained from the health-care provider interviewed, and survey was completed in private rooms to ensure privacy. Data used for the study were stored in a secured folder with limited access.

## Results

In total, 1141 maternal health-care providers were included in the original study. Seven anesthetists were dropped from the analysis since they constitute only 0.6% of the sample; too few to permit statistical inference. The final analytical sample included 1134 health-care providers working in 154 facilities, including 627 providers in Uganda and 507 in Zambia (Table 1).

**Table 1:** Characteristics of health-care providers, facilities and context in Uganda and Zambia

	Total	Uganda	Zambia
	(*n* = 1134)	(*n* = 627)	(*n* = 507)
	*n* (%)	*n* (%)	*n* (%)
Provider outcomes			
Satisfied with the job^a^			
Strongly agree	589 (52.3%)	291 (46.8%)	298 (59.0%)
Somewhat agree	363 (32.2%)	212 (34.1%)	151 (29.9%)
Somewhat disagree	102 (9.1%)	75 (12.1%)	27 (5.3%)
Strongly disagree	73 (6.5%)	44 (7.1%)	29 (5.7%)
Intention to stay in the job^b^			
Strongly agree	481 (42.8%)	306 (49.4%)	175 (34.8%)
Somewhat agree	378 (33.7%)	201 (32.4%)	177 (35.2%)
Somewhat disagree	129 (11.5%)	62 (10.0%)	67 (13.3%)
Strongly disagree	135 (12.0%)	51 (8.2%)	84 (16.7%)
Provider characteristics			
Age (mean ± SD)	35.60 (10.37)	34.00 (10.25)	37.58 (10.19)
Days of training in past year (mean ± SD)	4.22 (9.62)	4.04 (9.82)	4.43 (9.38)
Female	775 (68.3%)	441 (70.3%)	334 (65.9%)
Cadre			
Nurse assistant^c^	117 (10.3%)	117 (18.7%)	0 (0.00%)
Enrolled nurse^d^	367 (32.4%)	175 (27.9%)	192 (37.9%)
Enrolled midwife^e^	226 (19.9%)	106 (16.9%)	120 (23.7%)
Registered nurse^f^	139 (12.3%)	54 (8.6%)	85 (16.8%)
Registered midwife^g^	114 (10.1%)	65 (10.4%)	49 (9.7%)
Clinical officer^h^	126 (11.1%)	82 (13.1%)	44 (8.7%)
Doctor^i^	45 (4.0%)	28 (4.5%)	17 (3.4%)
Facility characteristics			
Hospital^j^ (ref. health center^k^)	532 (46.9%)	294 (46.9%)	238 (46.9%)
Public (ref. private^l^)	874 (77.1%)	453 (72.2%)	421 (83.0%)
Work environment^m^: inputs			
Functional equipment	727 (64.2%)	415 (66.3%)	312 (61.5%)
Adequate staff	453 (40.0%)	311 (49.7%)	142 (28.1%)
Satisfied pay	396 (35.4%)	230 (37.2%)	166 (33.1%)
Work environment^m^: management			
Adequate clinical supervision	917 (81.4%)	512 (81.9%)	405 (80.8%)
Manageable workload	491 (43.3%)	304 (48.5%)	187 (36.9%)
Adequate continuing education	742 (65.8%)	426 (68.3%)	316 (62.8%)
Opinions are respected in workplace	1008 (89.4%)	554 (88.9%)	454 (90.1%)
Context			
Urban	675 (59.5%)	347 (55.3%)	328 (64.7%)
Intervention	688 (60.7%)	376 (60.0%)	312 (61.5%)
Zambia	507 (44.7%)	0 (0.00%)	507 (100.0%)

Note:

a Satisfied with the job was assessed using the question: ‘In general, I am satisfied with this job.’

b Intention to stay in the job was assessed using the question: ‘If it were up to me, I would continue to work for this hospital/clinic for quite some time.’ Both questions were measured on a 4-point Likert scale—strongly disagree, somewhat disagree, somewhat agree and strongly agree.

c Nurse assistants are trained for about 6 months and exist only in Uganda.

d Enrolled nurses and

e enrolled midwives are trained for 2–3 years. Enrolled nurses are similar to licensed practical nurses in the United States.

f Registered nurses and

g registered midwives receive medical training for 3–4.5 years.

h Clinical officers receive 3 years of training.

i Doctors are typically trained for 5–7 years. Provider qualification can vary by country.

j Hospitals are equipped to provide comprehensive obstetric and neonatal care (CEmONC), which perform the seven basic functions of basic emergency obstetric and newborn care (BEmONC) and two additional services: cesarean delivery and blood transfusion.

k Health centers are equipped to provide BEmONC, which include seven signal functions: (i) administration of parenteral antibiotics, (ii) administration of uterotonic drugs for active management of the third stage of labor and prevention of postpartum hemorrhage, (iii) use of parenteral anticonvulsants for the management of preeclampsia/eclampsia, (IV) manual removal of placenta, (v) removal of retained products, (vi) assistance of vaginal delivery and (vii) basic neonatal resuscitation.

l Private facilities include both for-profit and not-for profit.

m For work environment variables, the questions were measured in a 4-point Likert scale—strongly disagree, somewhat disagree, somewhat agree and strongly agree. The percentage of providers who responded strongly agree and somewhat agree were included in the final analysis.

The mean age of providers was 35 years and the majority (68.3%) were female (Table 1). Enrolled nurses comprised the largest group of participants, followed by enrolled midwives. Most providers (77.1%) worked in publicly owned facilities and 59.5% were in urban areas.

Job satisfaction was higher than intention to stay: 52.3% of providers strongly agreed that they were satisfied with their jobs but only 42.8% strongly agreed that they would continue to work at their facility for quite some time.

Table 2 summarizes the results from the Shapley–Owen decomposition regression analysis. Among 1134 study participants, 1094 and 1087 had complete information for all covariates of interest for the final regression models for job satisfaction and intention to stay, respectively.

**Table 2:** Shapley–Owen decomposition analysis: variance attributed by the groups of explanatory variables in satisfied with the job and intention to stay in the job among health-care providers in Uganda and Zambia

	Satisfied with the job				Intention to stay in the job			
			*R* ^2^ decomposition (%)				*R* ^2^ decomposition (%)	
Covariate	Coefficient	95% CI	Individual	Group	Coefficient	95% CI	Individual	Group
Demographics								
Age	0.00	[−0.03, 0.03]	5.16	10.96	0.04	[−0.01, 0.08]	3.01	7.04
Age^2^	0.00	[0.00, 0.00]	5.54		0.00	[0.00, 0.00]	2.66	
Days of training in past year	0.00	[0.00, 0.01]	0.13		0.00	[0.00, 0.01]	1.04	
Female	−0.03	[−0.15, 0.09]	0.14		0.03	[−0.11, 0.17]	0.33	
Cadre (ref. enrolled nurse^a^)								
Nurse assistant^c^	−0.08	[−0.33, 0.16]	0.38	10.60	0.23*	[0.05, 0.41]	8.08	12.86
Enrolled midwife^b^	−0.02	[−0.17, 0.12]	1.07		−0.01	[−0.16, 0.14]	0.27	
Registered nurse^d^	−0.32**	[−0.50, −0.14]	5.41		−0.04	[−0.23, 0.15]	0.39	
Registered midwife^e^	−0.19	[−0.38, 0.03]	0.93		−0.23	[−0.51, 0.05]	1.81	
Clinical officer^f^	−0.24*	[−0.43, −0.05]	2.23		−0.21	[−0.43, 0.01]	1.35	
Doctor^g^	−0.25*	[−0.48, −0.01]	0.58		−0.29*	[−0.54, −0.05]	0.97	
Facility characteristics								
Hospital^h^ (ref. health center^i^)	0.03	[−0.11, 0.18]	0.24	0.34	0.05	[−0.10, 0.20]	0.43	0.80
Public (ref. private^j^)	−0.01	[−0.14, 0.11]	0.10		0.07	[−0.07, 0.21]	0.37	
Work environment^k^: inputs								
Functional equipment	0.09	[−0.03, 0.20]	5.22	31.12	0.02	[−0.11, 0.15]	2.06	20.56
Adequate staffing	0.16*	[0.04, 0.27]	5.33		0.13*	[0.01, 0.25]	7.40	
Satisfied with pay	0.33**	[0.23, 0.43]	20.57		0.23**	[0.10, 0.36]	11.10	
Work environment^k^: management								
Adequate clinical supervision	0.21**	[0.07, 0.36]	9.16	37.61	0.22*	[0.05, 0.39]	7.39	43.10
Manageable workload	0.04	[−0.07, 0.16]	3.66		0.25**	[0.14, 0.36]	14.05	
Adequate continuing education	0.17*	[0.04, 0.30]	7.52		0.09	[−0.02, 0.20]	4.14	
Opinions are respected in workplace	0.39**	[0.23, 0.56]	17.27		0.49**	[0.26, 0.73]	17.52	
Context								
Urban	0.04	[−0.11, 0.19]	0.34	9.37	−0.12	[−0.27, 0.03]	3.25	15.64
Intervention	−0.01	[−0.13, 0.12]	0.21		0.08	[−0.04, 0.21]	1.12	
Zambia	0.23**	[0.10, 0.36]	8.82		−0.29**	[−0.43, −0.15]	11.27	
Observations	1094				1087			
Full model *R*^2^	0.17				0.18			

Note:

** *P* < 0.01,

* *P* < 0.05,

95% CI: 95% confidence interval. Satisfied with the job was assessed using the question: ‘In general, I am satisfied with this job.’ Intention to stay in the job was assessed using the question: ‘If it were up to me, I would continue to work for this hospital/clinic for quite some time.’ Outcome variables are continuous variables and measured on four Likert-like scales. Standard errors are adjusted for clustering with facilities.

a Enrolled nurses and

b enrolled midwives are trained for 2–3 years. Enrolled nurses are similar to licensed practical nurses in the United States.

c Nurse assistants are trained for about 6 months and exist only in Uganda.

d Registered nurses and

e registered midwives receive medical training for 3–4.5 years.

f Clinical officers receive 3 years of training.

g Doctors are typically trained for 5–7 years. Provider qualification can vary by country.

h Hospitals are equipped to provide comprehensive obstetric and neonatal care (CEmONC), which perform the seven basic functions of basic emergency obstetric and newborn care (BEmONC) and two additional services: cesarean delivery and blood transfusion.

i Health centers are equipped to provide BEmONC, which include seven signal functions: (i) administration of parenteral antibiotics, (ii) administration of uterotonic drugs for active management of the third stage of labor and prevention of postpartum hemorrhage, (iii) use of parenteral anticonvulsants for the management of preeclampsia/eclampsia, (iv) manual removal of placenta, (v) removal of retained products, (vi) assistance of vaginal delivery and (vii) basic neonatal resuscitation.

j Private facilities includes both for-profit and not-for profit.

k For work environment variables, these questions were measured in a 4-point Likert scale—strongly disagree, somewhat disagree, somewhat agree and strongly agree. The percentage of providers who responded strongly agree and somewhat agree were included in the final analysis.

**Figure 1:**
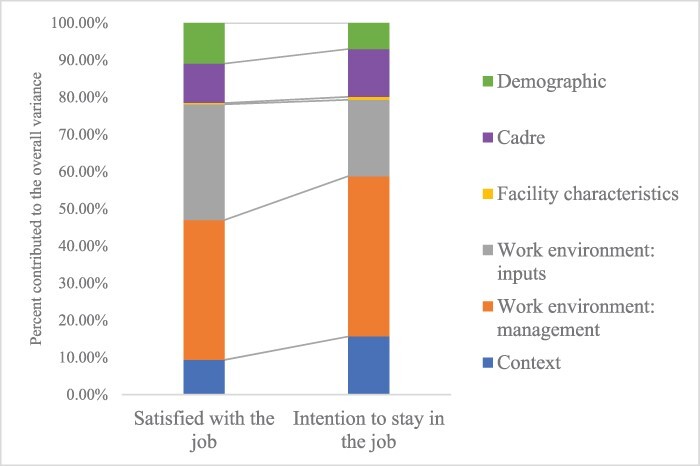
Contribution of explanatory variables to the explained variance in satisfied with the job and intention to stay in the job among health-care providers in Uganda and Zambia, *n* = 1134. Demographics included age, gender and on-site training. Cadre included seven types of providers based on the length of training: (i) nurse assistant, (ii) enrolled nurse, (iii) enrolled midwife, (iv) registered nurse, (v) registered midwife, (vi) clinical officer and (vii) general doctor, doctor specialist and medical licentiate. Facility characteristics included facility type and public vs. private ownership. Work environment: inputs category included three independent variables measuring provider’s opinion on the facility’s equipment, the level of staffing and whether they were satisfied with their current pay compared to similar jobs in other organizations. Work environment: management category included four independent variables measuring provider’s opinion on clinical supervision, whether their workload was manageable, whether their facilities provided adequate in-service (continuing) education to improve their clinical skills and whether they felt like their opinions were respected at work. Context covariates included urban/rural location, intervention/control districts and country (Uganda or Zambia).

Figure 1 shows the share of variance in job satisfaction and intention to stay explained by each group of covariates.

The overall model explained 17% of the variance in satisfaction and 18% of the variance in intention to stay in the job. Across the six groups of covariates, work environment (both management and inputs) explained the largest share of the explained variance in both outcomes. Management factors (clinical supervision, workload, continuing education and respect of opinions) explained 37.6% of the variance in satisfaction and 43.1% of the variance in intention to stay. The variable reflecting whether providers believed their opinions were respected in the workplace explained the largest share of the variance in intention to stay (17.5%) among the 22 individual factors.

Input-related covariates (functional equipment, staffing and pay) also explained an important share of the variance in both outcomes. In particular, being satisfied with the pay was the strongest contributor to variance in job satisfaction (20.6%).

In contrast, facility characteristics—ownership (public/private) and type (hospital/health center)—explained <1% of the variance in job satisfaction and intention to stay. We found no significant difference in job satisfaction among providers who worked in public and private facilities. Similarly, ∼40% of providers worked in the hospitals, and the health centers strongly agreed that they would continue to work at their facilities for some time.

Provider cadre explained 10.6% and 12.9% of the variance in satisfaction and intent to stay, respectively, and appeared to have an inverse relationship with providers’ intention to stay whereby higher trained providers were less likely to stay. More educated providers were less satisfied with their job than less educated providers: registered nurses reported 32 percentage points (95 CI −0.50, −0.14) lower job satisfaction than enrolled nurses. Registered nurses receive an additional 1–1.5 years of training compared to enrolled nurses. Similarly, doctors reported a significantly lower desire to stay in the job than enrolled nurses.

Provider demographics (age, gender and days of training in the past year) explained very little of the variance in both outcomes. Gender contributed <1% of the overall variance for job satisfaction and intention to stay.

Among the context covariates (urban area, intervention district and country), we found that country explained the largest share of variance in both outcomes (nearly 10%). A greater share of providers was satisfied in Zambia, but Ugandan providers were more likely to report that they intended to stay in their current job. Being in an urban area appeared negatively associated with intention to stay but explained very little of the variance in job satisfaction.

Correlations between the two outcome variables and the work environment covariates were generally low (<0.3) (**additional file 2**).

## Discussion

### Statement of principal findings

In this analysis, we used data from provider interviews to explore the factors associated with job satisfaction and intention to stay across multiple cadres of health-care providers who performed obstetric care in Uganda and Zambia. We found several factors associated with these outcomes.

The model explained 17–18% of the total variance in the two outcomes. This is consistent with other studies showing that job satisfaction is a complex construct affected by a multitude of factors, some not measured here, including personality trait and affective disposition [24, 25]. Other studies found similar overall variance explained [26, 27]. On the other hand, a study by Lee and colleagues explained 49% of variance in nurses’ job satisfaction in one province of Canada [28].

### Interpretation within the context of the wider literature

For the portion of the variance explained by the model variables, we found that work environment related to management explained most of the variation in both outcomes. Whether the providers feel that their opinions are being respected at their workplace was a much stronger driver of satisfaction than the type or ownership of the facilities. Other management efforts—availability of adequate continuing education and clinical supervision—were also significantly associated with job satisfaction. Other studies from sub-Saharan Africa similarly found that managers play a crucial role in keeping providers satisfied [29–32]. Intention to stay has also been strongly associated with quality of management in South Africa and Tanzania [5, 33, 34]. Educating managers on promoting competency, honesty and autonomy could aid in retaining satisfied health-care providers in sub-Saharan Africa.

Whether providers were satisfied with their pay was also a strong determinant, explaining 20.6% of the explained variance in job satisfaction. In low-income countries, a strong association between job satisfaction and salary has resulted in increased compensation and thus has proven to be a key strategy in improving satisfaction [35, 36]. However, in our study, two-thirds of providers reported dissatisfaction with their current pay. Frequent salary delays especially in the public sector have resulted in providers seeking for alternative sources of income or skipping work [37]. Thus, policymakers must attempt to meet the financial need of health-care providers in order to keep their satisfaction high.

A manageable workload (14.1%) and adequate number of staff (7.4%) also influenced providers’ intention to stay in the job. In a study from Senegal, midwives reported a heavy workload as a major reason to look for different job opportunities [7]. Adequate human resource is fundamental for retaining the health-care providers and ultimately influencing the care that patients receive. In Kenya and Malawi, women delivering in facilities with a higher ratio of clinical staff per maternity bed received more competent care [38]. Strategic scheduling of staff and managing workload should be part of the health management improvement plan.

Provider cadre was also an important determinant, and years of clinical education had an inverse relationship with intention to stay. More educated providers (doctors, clinical officers and registered nurses) appear much less likely to remain in their current jobs than enrolled midwives/nurses and nursing assistants. A similar finding was observed in a study in Uganda where doctors were more likely to report that they were eager to leave their job within 2 years compared to nurses and clinical officers [13]. This may be due to a greater number of job opportunities for better trained workers in other sectors. This is demonstrated in Ethiopia, where doctors have a higher chance of transitioning to other sectors such as universities and hospitals than nurses; thus, they are more inclined to leave their work [33]. Efforts to retain doctors and/or other higher-educated providers should be designed to ensure a functional health-care workforce.

Provider demographics, facility level and ownership were not associated with satisfaction and intention to stay. The share of variance in job satisfaction explained by work environment covariates (both management and input) was three times greater than that explained by provider demographics. Perhaps surprisingly, <1% of the variance was explained by facility characteristics—type (hospital/health center) and ownership (public/private). In studies from Uganda and Ethiopia, public and private sector workers, and hospital vs. health center staff had similar overall levels of job satisfaction [13, 39]. This demonstrates that determinants of job satisfaction are more correlated with individual-level needs such as interaction with supervisors, salary and quality of management than general facility characteristics. We also found important differences in both outcomes between Uganda and Zambia. In Zambia, a greater share of health providers was satisfied with their job but a lower share intended to stay compared to Uganda. This finding is surprising considering that the Zambian health system is better resourced than the Ugandan health system. Current health expenditure per capita and the number of nurses and midwives per 1000 people are higher in Zambia compared to Uganda [40]. However, when study participants were asked whether they felt there was an adequate number of providers in their facilities, a higher percentage of Ugandan providers (49.7%) strongly agreed with the statement compared to Zambian providers (28.1%). A similar finding was observed where South African providers reported lower satisfaction than either Malawi or Tanzania even though South Africa has much better health-care resources [41]. This observed difference between countries suggests that job satisfaction could also be influenced by other cultural, economic and political factors such as providers’ expectations and labor market conditions [41, 42].

### Strengths and limitations

This study has several limitations. First, our result is limited to health-care providers who performed obstetric care in Uganda and Zambia, which limits generalizability and may not apply to other clinical settings. Thus, interpretation of this study in relation to providers in other clinical settings and countries should be done with caution. Second, our sample only included a small number of doctors (*n* = 45, 4.0%); therefore, the results for this cadre should be interpreted with caution. It is common to find a low number of doctors especially in low- and middle-income countries. Third, nurse assistants exist only in Uganda. Thus, we assigned enrolled nurses as a reference group to be consistent across both countries. Fourth, our data were collected in 2013, and certain factors may have changed since. However, recent studies have found similar levels of job satisfaction and intention to stay in Uganda and Zambia [43, 44]. Therefore, we believe that our conclusions are still applicable and relevant to the study of provider retention in these countries. Lastly, the findings are applicable only to the study countries.

### Implications for policy, practice and research

Future studies should investigate providers in other clinical settings to aid the understanding of the factors contributing to providers’ intention to stay in their job and their job satisfaction. Also, more studies should be conducted across multiple provider cadres and countries to obtain a more comprehensive picture of the factors of job satisfaction and intention to stay. Studies have found a strong correlation between job satisfaction and quality of hospital care [45, 46]. Thus, the correlation between the quality of obstetric care and job satisfaction should be investigated to ensure mothers and newborns receive high-quality care.

## Conclusions

Health-care providers who worked in well-managed facilities were highly likely to stay and to feel satisfied about their jobs. Thus, this study emphasizes the role of health managers in improving the satisfaction and intention to stay. Provider satisfaction is highly correlated with the quality of care that patients received [45–47]. Thus, investment in health management systems is needed to maintain a satisfied health-care workforce so that high-quality care can be provided for patients in sub-Saharan Africa.

## Supplementary Material

mzab128_Supp

## Data sharing statement

The datasets used and/or analyzed during the current study are available from the corresponding author on reasonable request.
