# Supplementary material for: Predictors of job satisfaction and intention to stay in the job among health-care providers in Uganda and Zambia
Source: Int J Qual Health Care. 2021 Sep 9;33(3):mzab128. doi: 10.1093/intqhc/mzab128 (PMC8480544; doi:10.1093/intqhc/mzab128)
Supplement: mzab128_Supp [file mzab128_supp.zip › SMGL_satisfaction_additional file2.docx]

# Additional file 2 – An unadjusted correlations between the satisfied with the job and intention to stay in the job and the work environment covariates using Pearson’s correlation coefficient.

|  | Satisfied with the job | Intention to stay in the job | Functional equipment | Adequate staffing | Satisfied with pay | Adequate clinical supervision | Manageable workload | Adequate continuing education | Opinions are respected in work place |
| --- | --- | --- | --- | --- | --- | --- | --- | --- | --- |
| Satisfied with the job | 1.00 | -- | -- | -- | -- | -- | -- | -- | -- |
| Intention to stay in the job | 0.28 | 1.00 | -- | -- | -- | -- | -- | -- | -- |
| Functional equipment | 0.15 | 0.12 | 1.00 |  | -- | -- | -- | -- | -- |
| Adequate staffing | 0.13 | 0.18 | 0.19 | 1.00 | -- | -- | -- | -- | -- |
| Satisfied with pay | 0.22 | 0.19 | 0.15 | 0.19 | 1.00 | -- | -- | -- | -- |
| Adequate clinical supervision | 0.17 | 0.17 | 0.16 | 0.15 | 0.09 | 1.00 | -- | -- | -- |
| Manageable workload | 0.13 | 0.22 | 0.16 | 0.32 | 0.27 | 0.11 | 1.00 | -- | -- |
| Adequate continuing education | 0.16 | 0.15 | 0.19 | 0.19 | 0.09 | 0.21 | 0.15 | 1.00 | -- |
| Opinions are respected in work place | 0.22 | 0.23 | 0.17 | 0.12 | 0.15 | 0.17 | 0.12 | 0.20 | 1.00 |

Satisfied with the job was assessed using the question: ‘In general, I am satisfied with this job.’ Intention to stay in the job was assessed using the question: ‘If it were up to me, I would continue to work for this hospital/clinic for quite some time.’ Outcome variables are continuous variables and measured on four Likert-like scales. For work environment variables, these questions were measured in a four-point Likert scale - strongly disagree, somewhat disagree, somewhat agree, and strongly agree. The percentage of providers who responded strongly agree and somewhat agree were included in the final analysis.
